# With or Without You? People Feel Less Autonomous During Social Interactions, Except With Close Others

**DOI:** 10.1177/01461672251378784

**Published:** 2025-10-16

**Authors:** Elaine Hoan, Geoff MacDonald, Jessie Sun

**Affiliations:** 1 University of Toronto, Ontario, Canada; 2 Washington University in St. Louis, MO, USA

**Keywords:** social interaction, autonomy, solitude, well-being, romantic relationships

## Abstract

Social interaction bolsters well-being and relatedness. However, less is known about costs of social interaction, such as loss of autonomy. Here, we test a potential autonomy–relatedness tradeoff. College student participants completed experience sampling method self-reports (*N* = 352, 10,046 observations) of their social interactions, feelings of social connectedness, autonomy, and positive affect in the past hour. Participants reported lower autonomy when socially interacting compared with being alone. This was especially true for people with higher levels of attachment avoidance. Crucially, interaction partner matters: Compared with being alone, people report lower autonomy when interacting with non-close others, similar levels of autonomy when interacting with friends and family, and higher autonomy when interacting with romantic partners. These findings provide ecologically valid evidence for an autonomy–relatedness tradeoff during social interactions with non-close others but show that interactions with romantic partners uniquely fulfill both autonomy and relatedness needs.

“Alone had always felt like an actual place to me, as if it weren’t a state of being, but rather a room where I could retreat to be who I really was.”

– Cheryl Strayed

Relatedness (also known as belongingness or connectedness) has been argued to be a fundamental human need (Baumeister & Leary, 1995; Dweck, 2017; Kenrick et al., 2010; Maslow, 1943). Having more social interactions is consistently associated with better well-being (Sandstrom & Dunn, 2014; Sun, Harris, & Vazire, 2020), whereas lack of social interaction is associated with increased loneliness and risk of mortality (Holt-Lunstad, 2021). But are social interactions unreservedly beneficial? Existing work suggests that social interactions have diminishing returns for well-being (Kushlev et al., 2018; Ren et al., 2022; Stavrova & Ren, 2021). This might be because in addition to benefits from social interaction, there are also well-being benefits associated with solitude. Although solitude is defined in various ways, we define it here as the absence of interaction with others, regardless of one’s physical proximity to others (Nguyen et al., 2025; Weinstein & Adams, 2025). One important function of solitude is to seek space for freedom and autonomy (Ren et al., 2024; Weinstein, Hansen, & Nguyen, 2023). Thus, existing literature implies a potential tradeoff: Social interactions satisfy relatedness needs at some degree of cost to autonomy. Here, we provide a direct empirical test of this relatedness-autonomy tradeoff and further examine the conditions under which this tradeoff is minimized or exacerbated.

## Autonomy and Relatedness: Conflicting or Complementary?

Autonomy and relatedness are two fundamental motives that appear in various taxonomies of basic psychological needs (Dweck, 2017; McLeod, 2007; Ryan & Deci, 2000; Talevich et al., 2017). According to Self-Determination Theory, autonomy describes the extent to which a person is self-governing, such that they can act with a sense of volition and willingness (Deci & Ryan, 2008). When the need for autonomy is frustrated, people feel pushed in an unwanted direction, and that they no longer “own” their motivations. Relatedness describes the experience of warmth, bonding, and care (Ryan & Powelson, 1991). When the need for relatedness is frustrated, people feel socially alienated and lonely.

Autonomy and relatedness needs can, in principle, be fulfilled simultaneously. For example, imagine that two friends, Gerald and Chandra, go on a hike. Both autonomy and relatedness needs could be met if Gerald and Chandra have a choice in which trail to hike (i.e., autonomy) while spending time together (i.e., relatedness). Given the importance of autonomy and relatedness for well-being (Reis et al., 2000), simultaneously fulfilling both needs might best promote individual flourishing (Sheldon & Niemiec, 2006). Indeed, autonomy and relatedness satisfaction is typically positively correlated (Heppner et al., 2008; Inguglia et al., 2015; Kasser & Ryan, 1999). For example, daily diary research shows that when participants felt like they were acting in line with their values, they reported more positive social interactions (Baker et al., 2017).

However, there are often situations where autonomy and relatedness are in tension (Bakan, 1966; Blatt & Luyten, 2009). Indeed, some models portray the fundamental motives of agency (promoting one’s own interests) and communion (promoting others’ interests) as being in tension with one another (Schwartz, 1992; Wiggins, 1991). For example, imagine that Chandra finds a new path she wants to explore, but Gerald would prefer the original trail. Gerald could prioritize relatedness by following Chandra onto the new path or prioritize autonomy by sticking to his preferred route (without Chandra). Thus, it is not always possible to do exactly what you want while also getting along with others.

Recent work has begun to test the autonomy–relatedness tradeoff using the Day Reconstruction Method, but results conflict depending on how social time and autonomy are assessed. Consistent with an autonomy–relatedness tradeoff, people report *less* autonomy satisfaction on days when they spent more time with others (Weinstein, Vuorre, et al., 2023). However, participants in that same study also reported how much autonomy they experienced in social versus solitude contexts each day (Adams & Weinstein, 2024). For example, the prompt for social contexts was “When you were interacting (interacting with others in-person or over technology), how true were these statements for you . . .” This context-specific assessment of autonomy showed that people reported slightly *greater* autonomy in social (*M* = 5.20 on a 7-point scale) versus solitude (*M* = 5.11) contexts. Importantly, neither of these approaches directly captures how much autonomy people experience *while* they are socially interacting (vs. not). The first approach correlates *day-level* social time with *day-level* autonomy. The second approach requires people to mentally aggregate across all of their social versus solitude contexts that day (rather than measuring how much autonomy people experienced during each episode of the day) and is thus potentially subject to retrospective biases and lay beliefs about how socializing affects autonomy. To more directly test whether there is a tradeoff between autonomy and relatedness, we use the Experience Sampling Method (ESM) to capture people’s momentary experiences of autonomy and relatedness while they are socially interacting (vs. not). We further examine whether this tradeoff varies depending on interaction partners and individual differences in attachment.

## Moderators of the Autonomy–Relatedness Tradeoff

### With Whom Does the Tradeoff Occur? The Role of Interaction Partners

Deci and Ryan (2000) note that autonomy and relatedness can be either complementary or antagonistic, depending on the circumstances. For example, autonomy and relatedness might conflict more in certain relationships. A person may experience less conflict between autonomy and relatedness when interacting with close others with whom they are comfortable expressing their preferences (Hudson et al., 2020; Ruan et al., 2020; Sprecher et al., 2013). For example, if Gerald and Chandra are old friends with a history of trust, Gerald could express that he wants to stay on the main trail with greater ease. Indeed, Downie et al.’s (2008) study found that participants felt the most autonomous (i.e., like they could be themselves) in their interactions with friends and family compared with interactions with coworkers or acquaintances. However, closeness may not eliminate the conflict. Chandra could remain persistent in her desire to explore this new path even if Gerald voices his preferences.

### For Whom Does the Tradeoff Occur? The Role of Attachment

Some people might also have a greater tendency to perceive a tension between autonomy and relatedness. For example, people who more strongly value one need over the other may experience greater conflict between the two. Attachment theory offers a useful framework for understanding these individual differences, as it describes how people might balance independence and closeness in their relationships (Mikulincer et al., 2003). Attachment avoidance is characterized by relatively high valuation of autonomy (e.g., a strong desire for independence) and relatively low valuation of relatedness (e.g., discomfort with intimacy and distrust in others; Mikulincer et al., 2003). If Chandra is high in attachment avoidance (“avoidantly attached,” for shorthand), she may feel that sticking to the main route would come at an unacceptable cost to her autonomy and choose the new path to maximize her autonomy at the cost of relatedness. In contrast, if Chandra is less avoidantly attached, she might be more willing to compromise and stick to the main path to spend more time with Gerald, without perceiving this as a major autonomy threat. Consistent with this idea, Hadden and colleagues (2015) found that more avoidantly attached undergraduate students reported lower autonomy support in their romantic relationships. Similarly, Girme and colleagues (2019) found that people who were more avoidantly attached experienced lower levels of autonomy during conversations with one’s romantic partner.

Likewise, those who value relatedness more than autonomy could also find it more difficult to balance the two needs. Attachment anxiety is characterized by relatively strong but unfulfilled relatedness needs which manifest as fear of rejection, difficulty being alone, and clinginess in relationships (Knoke et al., 2010; Spielmann et al., 2013). If Gerald is anxiously attached, he may decide to follow Chandra onto the new path to avoid conflict and maximize their bonding time. In contrast, if Gerald is less anxiously attached, he might express his preference and stick with the original trail, knowing that Chandra will still like him. People who have high levels of anxious attachment have been shown to put others’ needs above their own at times. For example, Impett and Gordon (2010 ) demonstrated in a 14-day daily diary that more anxiously attached participants were more likely to engage in sacrifice, especially to gain recognition from their romantic partner. Importantly, this overemphasis on relatedness could negatively impact the fulfillment of anxiously attached individuals’ autonomy needs. Indeed, Hadden et al. (2015 ) found that anxious attachment was associated with lower autonomy need fulfillment.

## The Present Study

Altogether, although social interactions have obvious benefits for fulfilling relatedness needs, a growing literature suggests that people also have good reasons for sometimes seeking solitude. Here, we directly test whether social interactions present a tradeoff between two fundamental needs—autonomy and relatedness. We further explore whether the extent of this tradeoff depends on the interaction partner and one’s attachment style. To our knowledge, no research has empirically examined the conditions under which the presence versus absence of social interactions satisfies relatedness versus autonomy needs. To address this gap, we analyzed experience sampling reports of connectedness and autonomy when participants were socially interacting (vs. not) across 14 days.

## Method

### Ethics and Open Practices Statement

We used data from the first wave of the longitudinal Personality and Interpersonal Roles Study (PAIRS). Data collection and study procedures were approved by Institutional Review Boards at Washington University in St. Louis (IRB ID: 202312081). A data transfer agreement was approved by the Research Ethics Board at the University of Toronto (Protocol #: 44839). The ESM social interaction and affect variables have been used in other published manuscripts (Weidman et al., 2020; Wilson et al., 2015, 2016; Sun, Harris, & Vazire, 2020; Sun, Schwartz, et al., 2020; see https://osf.io/3uag4/wiki/home/ for a full list of studies using PAIRS). The most closely related paper examined the associations between the presence and quality of social interactions (specifically, conversational depth and self-disclosure) with momentary happiness and connectedness (Sun, Harris, & Vazire, 2020). To facilitate effect size comparisons with autonomy, we report the effects of the presence of social interactions on momentary happiness and social connectedness, which have previously been reported (Sun, Harris, & Vazire, 2020). However, the current paper uses a larger subset of participants than the previous paper (which included a smaller subset of participants who had audio recordings; Sun, Schwartz, et al., 2020). All analyses involving the autonomy, relationship status, romantic versus non-romantic interaction partner, and attachment style variables are novel.

The original goals of the project were to explore differences between the social experiences of single and partnered individuals. However, we decided after initial analyses that the college student population would not be ideal for testing these ideas. Relationship status is more transient at this life stage and college students are in a unique social context where they can engage in social interactions regardless of relationship status. As such, relationship status would be a less meaningful variable among college students compared to midlife and older adults. For these reasons, we did not want to draw strong conclusions about single versus partnered individuals’ social lives based only on college students. However, our exploration of these data along themes of relatedness and autonomy led to the current exploratory analyses. In addition, because of our original interest in the social experiences of single and partnered individuals, we initially focused on comparing romantic versus non-romantic interactions. However, additional analyses that further distinguished between different types of non-romantic others—namely, close (i.e., friends and family) versus non-close others—were incorporated in response to reviewer feedback.

None of the analyses conducted in the current study were pre-registered because data collection occurred years ago and one of the co-authors was already familiar with the dataset from previous projects. Therefore, all results are exploratory. The data and R code needed to reproduce this paper’s analyses are available at https://osf.io/bqaxn/.

### Participants

The PAIRS participants were 434 undergraduate students from Washington University in St. Louis recruited from 2012 and 2013 using flyers and classroom announcements across campus. Participants were compensated $20 for an initial laboratory-based assessment and entered into a lottery with the chance of winning $100 for completion of ESM surveys. Completion of all ESM surveys resulted in a 1 in 10 chance of winning the lottery. Participants also received a “time capsule” with feedback on their personality changes across the seven waves of the study. Data collection ended at the end of the semester when at least 400 participants were recruited.

The current study used a final subset of 352 participants (238 women, 113 men, 1 gender not reported) after exclusions (see section “Data Exclusions”). Participants were ages 18 to 32 (*M* = 19.33 years, *SD* = 1.99) and they identified as Caucasian (*n* = 193), Asian (*n* = 83), Black (*n* = 39), American Indian or Alaska Native (*n* = 2), other or multiple races (*n* = 27) or did not disclose their race (*n* = 8).

### Procedure

Below, we describe the measures and procedures used for the present study. Codebooks for all measures in the larger study are available at https://osf.io/akbfj/.

### Measures

#### Baseline Measures

##### Relationship Status

To measure relationship status, participants were asked two different versions of a relationship status question. An initial batch of participants (77 out of our subset of 352 participants) were asked: “What is your current romantic relationship status?” (original item). Response options included being “in an exclusive romantic relationship,” “in a non-exclusive dating relationship,” “in a friends-with-benefits relationship,” “not romantically involved with anyone (single),” and “other.” After collecting this initial batch of participants, this question was updated for clarity. Specifically, participants in the later batch (275 out of our subset of 352 participants) were asked: “Are you currently in a romantic relationship?” (updated item). Response options included “Yes, I am in a romantic relationship” and “No, I am not in a romantic relationship.” If participants responded that they were in a romantic relationship, they were further asked about their romantic relationship status with the same response options as before, except for the “not romantically involved with anyone” option.

For analyses involving the romantic relationship variable, we included only participants who could be cleanly categorized as single or in a relationship. We categorized participants as being in a relationship (i.e., “partnered”; *n* = 231) if they reported being in an exclusive romantic relationship in either question and categorized them as being single (*n* = 121) if they were not romantically involved with anyone (original item) or not in a romantic relationship (updated item). Those with an ambiguous relationship status (*n* = 29; i.e., non-exclusive relationship, friends-with-benefits, other, missing response) were excluded from analyses involving relationship status.

##### Attachment Security

To capture individual differences in attachment security, participants completed the 9-item Relationship Structures Questionnaire (ECR-RS; Fraley et al., 2011), which was originally adapted from the Experiences in Close Relationships Questionnaire–Revised (ECR-R; Sibley et al., 2005). This scale includes a 6-item measure of attachment avoidance (e.g., “I talk things over with the important people in my life”; *ω* = .88) and a 3-item measure of attachment anxiety (e.g., “I’m afraid that the important people in my life may abandon me”; *ω* = .89). Participants responded to items on a 7-point scale (1 = *Strongly disagree*; 7 = *Strongly agree*).

#### ESM Measures

After completing the laboratory-based assessment, participants began the ESM (Larson & Csikszentmihalyi, 1983) portion of the study. Four times a day (12 p.m., 3 p.m., 6 p.m., and 9 p.m.) for 14 consecutive days, participants received a text message notification and were emailed a link to a survey about their experiences in the hour that preceded the notification (11 a.m.–12 p.m., 2 p.m.–3 p.m., 5 p.m.–6 p.m., and 8 p.m.–9 p.m.).

##### Presence of Interactions

In each ESM survey, participants were asked: “During the last hour, were you interacting with other people?” (response options: no, 1 person, 2 people, 3–5 people, more than 5 people). We recoded this item into a binary variable capturing whether a social interaction had occurred (coded as 1) or not (coded as 0) during the last hour. As previously mentioned, we define solitude as the absence of interaction with others, regardless of whether other people are physically present. However, the ESM survey also included an item that assessed whether participants were “Completely alone” (coded as 0) or “Around others” (coded as 1) in the past hour. In practice, presence of interactions was strongly correlated with the presence of others (within-person *r* = .64, *p* < .001). At times, we therefore use “alone” as shorthand for moments in which social interaction was absent.

##### Social Interaction Partners

If participants reported that they interacted with at least one person, they were then asked whether they had interacted with a family member, romantic partner, local best friend, and/or another close friend during the last hour, using a checklist that allowed them to select as many categories as applicable. We used the presence of interaction and social interaction partner variables to create four new variables that respectively capture interactions with only non-romantic others, two subtypes of non-romantic others—only friends/family and only non-close others—and only romantic partners (vs. no interactions at all). Table 1 shows the definitions of these four variables.

**Table 1.** Definitions of Interaction Partner Variables.

Interaction type	*n*	1 = participant indicated that they had a social interaction *and*:	Observations excluded (coded as NA)
Non-romantic others	9,133	Romantic partner was *not* selected	Romantic partner was selected
Friends/family	6,407	Only family member, local best friend, and/or another close friend was selected	Romantic partner was selected; or no specific interaction partners were selected
Non-close others	5,434	No specific interaction partners were selected	Any specific interaction partners were selected
Romantic partners	1,333	Only romantic partners selected	Family member, local best friend, and/or another close friend were selected; no specific interaction partners were selected; observations from single (i.e., unpartnered) participants

*Note*. For all interaction partner variables, 0 = no interactions at all. “Specific interaction partners” refers to the four checklist options: romantic partner, family member, local best friend, or another close friend.

Note that because the checklist did not comprehensively measure all possible types of interaction partner types (e.g., coworkers and strangers were not measured), some of the “romantic partner” interactions may have included a mix of romantic partners and non-romantic others who were not family, local best friends, or other close friends. Likewise, some of the “friend/family” interactions may have included a mix of friends/family and non-close others. As such, these two variables are best thought of as capturing “primarily” romantic partner or friend/family interactions, respectively.

To examine the effects of interactions with only romantic partners versus no one at all, we focused on the subset of 121 participants (*n* = 1,333 observations) who were in relationships. Although single participants occasionally reported interactions with romantic partners (*n* = 43 observations), we did not include them in analyses involving this variable because non-committed romantic partner interactions are likely different than committed romantic partner interactions.

##### Autonomy

To capture momentary feelings of autonomy, participants were asked, “In this situation, were you free to behave however you wanted?” using a 5-point scale (1 = *Not at all*, 5 = *Very much*). This measure conceptually aligns with dominant theories that define autonomy as a sense of volition and intention in one’s actions (Deci & Ryan, 2008). This measure is also similar to other one-item state measures of autonomy used in previous ESM studies (Johansen et al., 2024; e.g., “I felt a sense of choice and freedom in the things I did”; Wang & Hwang, 2020; e.g., “Regarding what I’m doing now, I feel I am doing what I want and what really interests me”), as well as commonly used trait measures of autonomy, such as the Basic Psychological Needs Scales (Deci & Ryan, 2000; e.g., “I feel like I am free to decide for myself how to live my life”; “I feel like I can pretty much be myself in my daily situations”).

That being said, this particular item has not previously been validated as a measure of autonomy. We therefore conducted a pre-registered follow-up ESM study to psychometrically validate our one-item measure against the three-item autonomy subscale from the Basic Psychological Needs Scales (BPNS; La Guardia et al., 2000), which has been used in previous daily life studies (Adams & Weinstein, 2024; Weinstein, Vuorre, et al., 2023). Providing strong evidence for the convergent validity of our one-item measure, this psychometric validation study showed that our one-item measure correlates strongly with the established three-item autonomy composite (within-person *r* = .60) and loads strongly onto the same latent factor as these three established items (for details, see Supplemental Material, Section 1).

##### Connectedness

To capture momentary feelings of connectedness as an indicator of relatedness, participants answered the question, “During the last hour, did you feel ‘close, connected’ to others?” using a 5-point scale (1 = *Not a lot*, 5 = *Very much*). This measure conceptually aligns with SDT understandings of relatedness as feelings of closeness and bonding (Ryan & Powelson, 1991).

##### Positive Affect

To capture momentary feelings of positive affect, participants answered the following two questions, which were aggregated to create a positive affect composite: “How much positive emotion did you experience?” (1 = *None at all*, 5 = *A lot*) and “During the last hour, how happy were you?” (1 = *Not at all*, 5 = *Very*; ω_WP_ = .83; ω_BP_ = .97). All participants had data on the happiness item, but data on the positive emotion item were missing for 85 of the 352 participants, as this item was added after data collection had begun.

### Data Exclusions

In line with exclusion criteria applied in previous papers that used the PAIRS ESM data (e.g., Finnigan & Vazire, 2018; Sun, Harris, & Vazire, 2020; Wilson et al., 2016), we excluded ESM reports (a) if they were completed more than 3 hours after the notification was sent, (b) if participants completed fewer than 75% of the items, (c) if participants used the same response option for at least 70% of the items, or (d) if participants indicated that they were asleep during the entire target hour. We also excluded practice ESM surveys that were completed during the participant’s initial laboratory session. Finally, we excluded participants who had fewer than five valid ESM reports. After applying these exclusions, the final analyses comprised 10,046 observations from 352 participants (*n*_partnered_ = 121, *n*_single_ = 231), with a mean of 28.54 observations (*SD* = 14.89) per participant.

### Data Analysis

All analyses were conducted in RStudio (Version 4.3.1; R Core Team, 2020). The data had a multi-level structure, with observations (Level 1) nested within participants (Level 2). We used multilevel modeling using the *lmer* package in R (Bates et al., 2015) to model the within-person associations between autonomy, social interactions, and attachment. All multilevel models included random slopes and used person-mean-centered predictor variables.

To compute standardized effects for multilevel regression coefficients, we applied the following formula when both predictors were continuous:

$$\beta =b*{\mathrm{SD}}_{\mathrm{wp}X}/{\mathrm{SD}}_{\mathrm{wp}Y}$$

where β = the standardized regression coefficient, *b* = the unstandardized regression coefficient, SD_wp*X*_ = the average within-person standard deviation of the predictor variable, and SD_wp*Y*_ = the average within-person standard deviation of the outcome variable.

When the predictor was a binary variable (e.g., the presence vs. absence of social interactions) and the outcome was continuous, we only standardized against Y, by applying this formula:

$$\beta =b/{\mathrm{SD}}_{\mathrm{wp}Y}$$

The attachment avoidance and anxiety variables were standardized (between persons) before being entered into analyses.

## Results

### Descriptive Statistics

Tables 2 and 3 present means, standard deviations, and intercorrelations among study variables.

**Table 2.** Descriptive Statistics of Social Interaction Variables Across All Participants and Split by Relationship Status.

Interaction type	All			Single			Partnered		
	Total count	% of total	*M* (*SD*) across participant	Total count	% of total	*M* (*SD*) across participants	Total count	% of total	*M* (*SD*) across participant
No Interactions	2,708	27%	.27 (.44)	1,805	18%	.27 (.45)	903	9%	.26 (.44)
Any Interactions	7,336	73%	.73 (.44)	4,770	47%	.73 (.45)	2,566	26%	.74 (.44)
Non-romantic other	6,425	64%	.70 (.46)	4,727	47%	.72 (.45)	1,698	17%	.65 (.48)
Family	772	8%	.08 (.27)	521	5%	.08 (.27)	251	3%	.07 (.26)
Friend	3,707	37%	.37 (.48)	2,554	25%	.39 (.49)	1,153	11%	.33 (.47)
Non-close other	2,726	27%	.27 (.44)	1,891	19%	.29 (.45)	835	8%	.24 (.43)
Romantic partner	911	9%	.09 (.29)	43	0%	.01 (.08)	868	8.64%	.25 (.43)

*Note.* Because the interaction partner categories were not mutually exclusive (e.g., a participant was able to report interacting with both a romantic partner and a close friend), the counts for the interaction partner categories do not add up to the total number of interactions. We recoded participants as having interacted with a “friend” if they said that they interacted with a “local best friend” and/or “another close friend” (in line with Wilson et al., 2015). We recoded participants as having interacted with a “non-close other” if they reported a social interaction but did not select any of the interaction partner types (i.e., romantic partner, family, local best friend, or another close friend) in the checklist. Means and standard deviations were computed on means for each participant (such that all participants were equally weighted in the calculation).

**Table 3.** Descriptive Statistics and Between-Person Correlations Among all Observed Variables.

Variable	Descriptive statistics				Between-person correlations				
	*M*	1–*ICC*(1)	*SD_WP_*	*SD_BP_*	1.	2.	3.	4.	5.
1. Presence of Social Interaction (ESM)	.74	.89	0.42	0.17					
2. Autonomy (ESM)	3.53	.89	1.18	0.49	−.01				
3. Connectedness (ESM)	2.86	.80	1.12	0.62	.54	.18			
4. Positive Affect (ESM)	3.47	.75	0.82	0.49	.34	.35	.61		
5. Attachment Avoidance (Trait)	2.71	–	–	1.32	−.09	−.17	−.39	−.32	
6. Attachment Anxiety (Trait)	3.11	–	–	1.79	−.12	−.11	−.23	−.25	.24

*Note. SD_WP_* = average within-person standard deviation; *SD_BP_* = between-person standard deviation. *ICC*(1), the intraclass correlation, represents the proportion of total variance (σ^2^_BP_+σ^2^_WP_) that is due to between-persons variability (σ^2^_BP_; i.e., mean-level differences on a variable across the 2 weeks), so 1–ICC(1) denotes the % of total variance due to within-person variability (σ^2^_WP_; i.e., fluctuations around a person’s mean level). Between-person correlations are based on the aggregate observed mean for each person. Correlations >|0.06| are significant at *p* < .05.

### People Feel More Connected but Less Autonomous During Social Interactions

**Figure 1.**
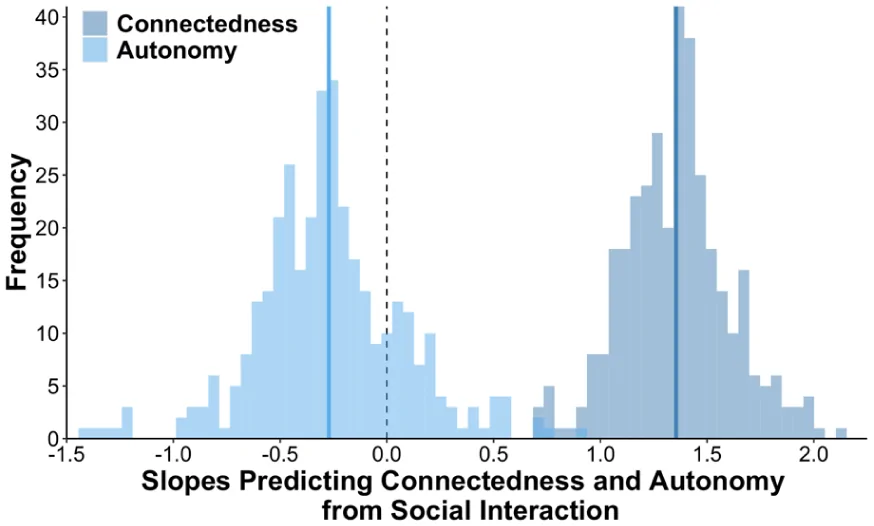
People Feel More Connected but Less Autonomous During Social Interactions. *Note.* This histogram shows the unstandardized within-person associations between momentary connectedness or autonomy and whether or not participants interacted with someone in the past hour. The solid vertical lines show the mean slopes.

As shown in a previous analysis of a subset of this dataset (Sun, Harris, & Vazire, 2020), participants felt more socially connected when they were interacting with at least one person compared with when they were not (β = 1.21, 95% confidence interval [CI] = [1.15, 1.26], *p* < .001; see Figure 1). Here, we considered one *cost* of social interaction: reduced autonomy. As shown in Figure 1, across all interaction partners, participants reported less autonomy when interacting with at least one person compared with when they were not (β = −0.23, 95% CI = [−0.30, –0.16], *p* < .001). Notably, the extent to which participants report a reduction in autonomy appears smaller than the gain in social connection. We interpret this reduction in autonomy as a “cost” of social interaction, because participants felt less positive affect in moments in which they felt less autonomous (β = 0.33, 95% CI = [0.30, 0.36], *p* < .001). In contrast, participants felt more positive affect in moments in which they felt more socially connected (β = 0.55, 95% CI = [0.52, 0.57], *p* < .001).

### Moderators of the Autonomy-Related Costs of Social Interaction

Next, we explored factors that might mitigate or amplify the autonomy-related costs of social interactions.

#### People Feel Less Autonomous With Non-Close Others, but More Autonomous With Romantic Partners

Does it matter who the interaction partner is? Interactions with close others (e.g., romantic partners, friends, and family) might be associated with a stronger decrease in autonomy as people feel pressure to accommodate their needs. Alternatively, interactions with close others might facilitate greater autonomy because people feel more comfortable being themselves and expressing their needs.

##### Non-Romantic Others

First, we estimated the effect of interacting with only non-romantic others (vs. no interactions at all) across the entire sample (*n* = 9,133 observations). Participants reported less autonomy when interacting exclusively with non-romantic others compared with when they were not interacting at all (β = −0.29, 95% CI = [−0.37, –0.22], *p* < .001). This was similarly true for single people (β = −0.30, 95% CI = [−0.39, –0.20], *p* < .001) and for those in relationships (β = −0.28, 95% CI = [−0.39, –0.17], *p* < .001), with no significant moderating effect of relationship status (β = −0.00, 95% CI = [−0.15, +0.15], *p* = .987).

**Figure 2.**
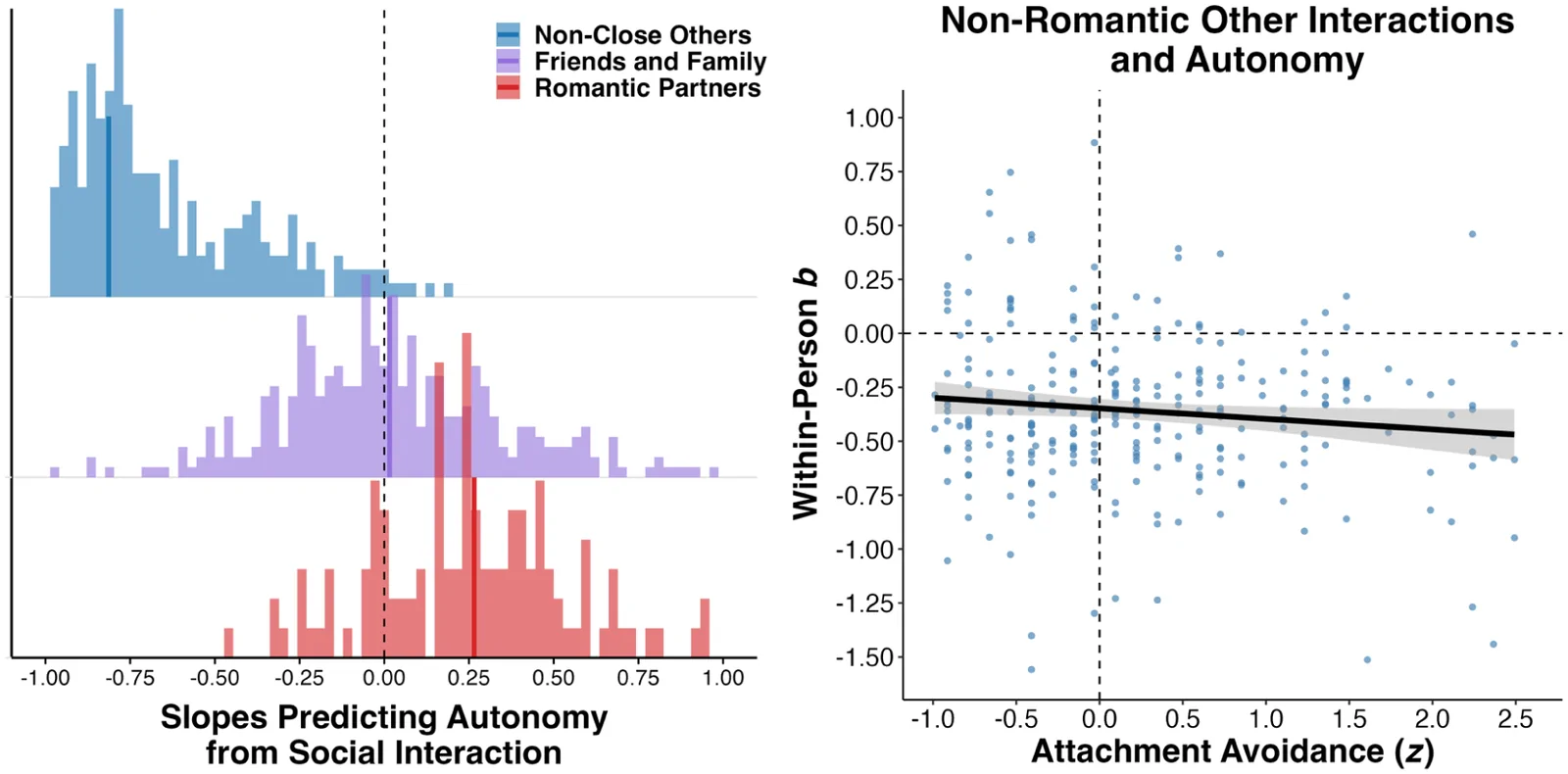
Moderating Effects of Interaction Partner and Avoidant Attachment Style. *Note*. Left: These histograms show the unstandardized within-person associations between interactions with either primarily romantic partners, friends, and family or non-close others (compared with not interacting with anyone) in the past hour and autonomy in the past hour across participants who were in relationships. The solid vertical lines show the mean slopes. Right: Predicted unstandardized within-person associations between interactions with exclusively non-romantic others (vs. no interactions) and autonomy in the past hour (solid black line) at different levels of trait attachment avoidance (*z*-scored), controlling for trait attachment anxiety, across all participants. Gray ribbons show 95% confidence intervals. Each point represents the within-person association for each person (extracted from multilevel model estimates).

Based on reviewer feedback, we conducted additional analyses to further clarify this finding. Specifically, our original approach may obscure important distinctions between close (e.g., friends and family) versus non-close (e.g., strangers and acquaintances) non-romantic others. Indeed, follow-up analyses across the entire sample showed that participants reported less autonomy when they exclusively interacted with non-close others compared with when they were not interacting at all (β = −0.69, 95% CI = 0.77, –0.61], *p* < .001; *n* = 5,434 observations; see Figure 2). However, participants reported no significant differences in autonomy in moments when they primarily interacted with friends and family compared with moments when they were not interacting at all (β = 0.01, 95% CI = [−0.07, +0.09], *p* = .741; *n* = 6,407 observations; see Figure 2). These results suggest that the apparent autonomy costs of interacting with non-romantic others only apply to *non-close* others. In contrast, we find no detectable autonomy costs of primarily interacting with friends and family, compared with being alone (see Supplemental Table S2 for additional comparisons between interaction partners).

##### Romantic Partners

Next, focusing on the subset of 121 participants (*n* = 1,333 observations) in relationships, we compared moments where participants primarily interacted with a romantic partner with moments in which they interacted with no one (coded as 0; *n* = 903 observations). Recall that participants in relationships felt *less* autonomous when interacting exclusively with non-romantic others (β = −0.28; reported above), compared with not interacting at all. In contrast, as shown in Figure 2, partnered participants reported *more* autonomy when interacting primarily with their romantic partners (*β* = 0.23, 95% CI [0.06, 0.39], *p* = .010) compared with when they were not interacting at all. In other words, interactions with romantic partners are associated with a *boost* in autonomy (compared with being alone).

##### Summary

These results show that the association between social interactions and autonomy depends on who the interaction partner is. Compared with being alone, participants tend to feel less autonomous when interacting with non-close others, similarly autonomous when interacting primarily with friends/family, and more autonomous when interacting with their romantic partners.

### Avoidant People Experience a Greater Drop in Autonomy During Social Interactions

Next, we explored whether the negative within-person association between the presence of social interactions and autonomy was moderated by attachment anxiety and attachment avoidance (entered simultaneously into the model). Given that romantic and non-romantic partner interactions had opposite associations with autonomy (described above), we investigated the moderating role of attachment style separately for non-romantic versus romantic partner interactions.

First, we focused on interactions with non-romantic partners (across partnered and single participants). This model revealed a significant interaction between the presence of social interaction and attachment avoidance (β = −0.09, 95% CI [−0.17, –0.02], *p* = .011), such that those who were more avoidantly attached experienced a larger decline in autonomy when interacting with non-romantic others compared with being alone (see Figure 2). There was no significant moderating effect of attachment anxiety (β = −0.00, 95% CI = [−0.07, +0.07], *p* = .958).

We further examined whether attachment style might moderate our finding that partnered participants reported greater autonomy when interacting exclusively with romantic partners compared with not interacting at all. Both anxious attachment (β = −0.11, 95% CI = [−0.32, +0.09], *p* = .273) and avoidant attachment (β = −0.14, 95% CI = [−0.32, +0.04], *p* = .139) showed trends toward smaller increases in autonomy when interacting with romantic partners (vs. not interacting at all), but neither interaction effect was significant. However, this analysis used only 1,313 observations, which limits our power to detect statistical interaction effects.

In sum, those who were higher in attachment avoidance showed a stronger autonomy-relatedness tradeoff, such that avoidantly attached participants reported even greater declines in autonomy when interacting with non-romantic others compared with being alone. There was no significant moderating effect of attachment anxiety. Finally, romantic partner interactions were associated with similar increases in autonomy regardless of attachment orientation.

### People Feel More Connected When They Feel More Autonomous During Social Interactions

So far, we have documented mixed costs and benefits of social interaction: People feel more connected but less autonomous when they are socially interacting (compared with when they are alone). This suggests a tradeoff between connectedness and autonomy at one level: Those who prioritize connectedness should engage in more social interactions, whereas those who prioritize autonomy might be inclined to avoid them. But does this imply that there is a general tradeoff between autonomy and connectedness?

To explore this, we examined within-person associations between autonomy and connectedness. We found a general positive association between connectedness and autonomy (β = 0.19, 95% CI = [0.16, 0.22], *p* < .001) such that greater feelings of momentary connectedness were associated with greater feelings of momentary autonomy across all everyday moments. Interestingly, there was a significant moderating effect of the presence of social interaction (β = 0.27, 95% CI = [0.22, 0.32], *p* < .001), showing that the positive within-person association between connectedness and autonomy was significant when participants were socially interacting (β = 0.38, 95% CI = [0.34, 0.41], *p* < .001) and was not significant when participants were not interacting (β = −0.04, 95% CI = [−0.12, +0.04], *p* = .30).

In sum, these results suggest that social interactions (vs. being alone) are, on average, associated with connectedness benefits and autonomy costs. But, in the specific context of being in a social interaction, people feel more connected *when* they feel more autonomous.

## Discussion

Well-being research generally gives the impression that social interaction is an unmitigated good. Here, however, we show that social interactions are associated with benefits for relatedness but sometimes incur costs for autonomy. Using ESM data, we found that people generally report experiencing less autonomy during social interactions compared with solitude. These autonomy costs were felt especially strongly by people who were more avoidantly attached. Crucially, interaction partner matters: Autonomy costs only applied to interactions with non-close others. Compared with being alone, people felt similarly autonomous when interacting primarily with friends/family but felt *more* autonomous when interacting with romantic partners.

The degree of situational control may best explain why social interaction is associated with less autonomy compared with being alone. For example, during social interactions, one might have to engage in sacrifices that subordinate one’s own personal goals for another (Righetti et al., 2020). In contrast, solitude affords much greater opportunities for autonomy than do social interactions. Consistent with this idea, qualitative work shows that people report seeking out solitude for freedom and autonomy (Ren et al., 2024; Weinstein, Hansen, & Nguyen, 2023). In line with SDT understandings of autonomy, a sense of self-reliance and an absence of pressure emerged as themes in people’s descriptions of their solitary experiences (Weinstein et al., 2021). However, as reviewed in the introduction, very few studies have quantitatively tested whether socializing is associated with autonomy costs and have found conflicting results based on different day-level assessments of social time and autonomy (Adams & Weinstein, 2024; Weinstein, Vuorre, et al., 2023). By capturing in-the-moment experiences of autonomy, our study overcomes retrospective biases and more directly demonstrates that people experience less autonomy when they are socially interacting versus not. Our findings both clarify conflicting results from previous work and further reveal for whom (avoidantly attached people) and with whom (non-close others) the autonomy–relatedness tradeoff occurs.

Importantly, however, we found that people report *greater* autonomy when they are primarily interacting with their romantic partners compared with when they are alone. This represents a reversal of our finding that people generally report less autonomy when they are socially interacting compared with when they are alone. However, the autonomy boost associated with romantic partner interactions is consistent with some existing work showing that people feel more autonomous during interactions with close others (friends and family) compared with interactions with less close interaction partners (coworkers and acquaintances; Downie et al., 2008). As Deci and Ryan (2014 ) posit, under “optimal conditions,” positive interrelations emerge between the basic psychological needs, such that feeling connected to others could facilitate feelings of autonomy (e.g., Patrick et al., 2007). Romantic relationships are often based on a foundation of communication and trust and typically contain unique affordances—such as sexual activity (Park & MacDonald, 2022)—through which important personal desires can be fulfilled while also satisfying relatedness needs. Our findings suggest that romantic relationships may offer a unique social context for simultaneously fulfilling relatedness and autonomy needs in the moment. This provides an important counterpoint to narratives of singlehood that emphasize the autonomy costs of having to coordinate decisions with a romantic partner (Kislev, 2024).

So far, we have conceptualized and measured autonomy as the freedom to behave and act. But the finding that close others are an exception to the general trend of lower autonomy during social interactions suggests that autonomy might be experienced in different ways. For instance, the philosopher Isaiah Berlin (2006) distinguished between two types of freedom: negative and positive. Negative freedom refers to the “freedom from” interference and constraints that prevent a person from acting as they choose, whereas positive freedom refers to the “freedom to” act according to one’s will and pursue one’s goals. We speculate that interactions with non-close others might restrict one’s negative freedom, making them feel that their options to act or behave are limited, whereas interactions with romantic partners, friends, and family could enhance peoples’ positive freedoms by providing the support and confidence needed to act on their freedoms. The idea that close others, including romantic partners, might facilitate positive freedoms is in line with attachment theory and research supporting romantic partners as a “secure base” with which individuals feel free to explore the world. For example, research has shown that receiving romantic partner support is predictive of autonomous behaviors such as perceived personal growth and successful goal attainment (Feeney, 2004; Fitzsimons & Bargh, 2003). As such, the autonomy that people feel with romantic partners may not just be fewer restrictions on behavioral choice, but also an imbued sense of freedom and ability to fully act on one’s desires.

Our data also revealed individual differences in the degree to which there was a tradeoff between autonomy and relatedness. Specifically, we found that avoidant people experienced a stronger decrease in autonomy during social interactions, consistent with existing work showing that highly avoidant individuals are more inclined to experience impediments to their autonomy (Girme et al., 2019; Hadden et al., 2015). This might be because avoidants see interaction partners as unresponsive and untrustworthy (Beck et al., 2014), and therefore less likely to meet their needs during social interactions. Future studies should continue to uncover characteristics of people who are more (vs. less) likely to experience an autonomy–relatedness tradeoff. One possible moderator is age. For example, in contrast to our findings, a 10-day ESM study showed that for older adults (mean age = 63 years), being accompanied by anyone was associated with greater satisfaction of both autonomy and relatedness needs compared with being alone (Wang & Hwang, 2020).

Although we find an autonomy–relatedness tradeoff when it comes to the choice between interacting or not, interestingly, autonomy and connectedness are positively associated *during* social interactions (but are not associated when people are alone). Two possible explanations are that (a) we feel more connected to those who provide us the space to be ourselves and that (b) we feel freer to express ourselves when we are more connected with others (consistent with the increase in autonomy when interacting with romantic partners). Moreover, whereas we interpret reduced autonomy as a “cost” of social interaction, it is important to note that the relationship between relatedness and positive affect was considerably stronger than the relationship between autonomy and positive affect. This finding suggests that for the average person, relatedness may play a stronger role than autonomy in shaping positive affect. However, future research could explore how individuals’ goals or motivations—such as the desire to feel connected versus independent—influence their social choices and, in turn, their momentary well-being when alone versus during social interactions.

We used a large ESM data set that captured real-time momentary feelings associated with recent social interactions. These data provide a strong, direct test of the autonomy-relatedness tradeoff associated with social interactions versus solitude in everyday life. However, our results should be interpreted in light of our study’s limitations. First, because participants were prompted with ESM assessments multiple times a day across several consecutive days, to reduce participant burden, the study only included a single-item measure of autonomy. Reassuringly, however, a psychometric validation study demonstrated that our one-item measure correlated strongly with another established ESM autonomy measure and loaded well on the same latent construct (for details, see Supplemental Material, Section 1).

Second, because the study only measured a limited set of interaction partners (i.e., romantic partners, family, and close friends), we were not able to fully isolate interactions that involved only one specific type of interaction partner. For example, “romantic partner interactions” capture interactions that involve romantic partners as well as unmeasured interaction partner categories (but not family or close friends). Considering that our results suggest that interactions with unmeasured (i.e., non-close) others are associated with lower autonomy, the main consequence of this inability to exclude unmeasured interaction partners is that our estimates of the autonomy benefits of interacting exclusively with a romantic partner (relative to being alone) are conservative. This limitation might also explain why we found no detectable boost in autonomy for interactions with friends/family (unlike Downie et al., 2008).

Third, because we collected data from a U.S. college student sample, it is unclear how our results would generalize to older populations or across cultures. For example, although we found that college students felt similarly autonomous when interacting with family/friends versus when they were alone, for college students, “family” interactions likely largely capture interactions with parents and siblings. It is therefore an open question as to whether parents would feel more or less autonomous when interacting with children—especially younger children—compared with being alone. If anything, considering that excessive social interaction can have null or even negative effects on one’s well-being (Kushlev et al., 2018; Stavrova & Ren, 2021), solitude may be especially appealing to parents, who often struggle to find “me-time” (Lévesque et al., 2020). In addition, it is possible that the autonomy–relatedness tradeoff may be particularly strong in the United States, which is a highly individualistic culture that places great value on autonomy (Inglehart & Oyserman, 2004). In contrast, people in collectivistic cultures that emphasize interdependence may not view social interaction as limiting autonomy as much (if at all).

Well-being research emphasizes the importance of social interaction for well-being. Consistent with this, our findings show that social interaction is largely a net gain for people. However, given that social interactions with non-close others often impose costs to autonomy, future research should continue to investigate when and why such autonomy costs emerge, as well as when they can be overcome. Such research may inform intervention strategies that balance sociability and solitude to fully meet people’s psychological needs.

## Supplemental Material

sj-docx-1-psp-10.1177_01461672251378784 – Supplemental material for With or Without You? People Feel Less Autonomous During Social Interactions, Except With Close Others Supplemental material, sj-docx-1-psp-10.1177_01461672251378784 for With or Without You? People Feel Less Autonomous During Social Interactions, Except With Close Others by Elaine Hoan, Geoff MacDonald and Jessie Sun in Personality and Social Psychology Bulletin
