# Supplementary material for: With or Without You? People Feel Less Autonomous During Social Interactions, Except With Close Others
Source: Pers Soc Psychol Bull. 2025 Oct 16;52(10):3336–48. doi: 10.1177/01461672251378784 (PMC13507498; doi:10.1177/01461672251378784)
Supplement: Supplementary file 1 [file sj-docx-1-psp-10.1177_01461672251378784.docx]

**1. Supplemental Psychometric Validation Study**

# Appendix A. Autonomy Psychometric Validation

We believe that our one-item autonomy measure, with its focus on behavioral freedom, conceptually captures a core part of the autonomy construct. However, to provide evidence for the convergent validity of this one-item measure, we conducted a follow-up psychometric validation study to validate this item against the more commonly used autonomy subscale of the Basic Needs Satisfaction Scale (La Guardia et al., 2000).

**Method**

***Ethics and Open Practices Statement***

Data collection procedures for this study were approved by the Institutional Review Board (IRB) at Washington University in St. Louis (Study Title: Personality and Daily Life; IRB ID: 202312081). We pre-registered our data collection procedure and analysis plan on AsPredicted (<https://aspredicted.org/xmcq-ym4d.pdf>). The data and code needed to reproduce these results are available in our OSF repository (<https://osf.io/bqaxn/?view_only=c4cf034aedb34a358839b52c162537d0>).

***Participants***

In total, participants were 26 undergraduate students from Washington University in St. Louis. Participants were asked to complete the study as part of an optional assignment for an undergraduate personality course and were compensated with course credit for their participation. Out of 26 students who signed up for the study, one did not provide consent for research use of their data, and three did not complete any ESM reports. The final sample comprised 22 participants who were aged 20.06 years old on average (range = 18 to 22, *SD* = 1.04) and who identified as female (*n* = 13), male (*n* = 7), and non-binary (*n* = 1), or preferred not to say (*n* = 1). Participants identified as White/Caucasian (*n* = 10), Asian (*n* = 5), Hispanic/Latino (*n* = 3), Multi-racial (*n* = 2), and Black/African American (*n* = 1), or did not disclose their race (*n* = 1).

***Procedure***

Participants received six surveys per day for six consecutive days (from Monday to Saturday) through SEMA 3 (O’Brien et al., 2024), a free smartphone app for collecting experience sampling data. Surveys were sent at 10am, 12:30pm, 3:00pm, 5:30pm, 8pm, and 10:30pm each day and expired 1 hour after they were sent. We originally pre-registered a plan to collect data across seven days, but due to human error, we failed to schedule surveys on the last day. The maximum number of reports per participant was therefore 36 (instead of 42). We had not preregistered a plan for dealing with partially complete reports, but we chose to exclude one report that had missing data for three of the four items of interest (as this report would have been excluded based on pairwise deletion in the correlational analyses below). After this exclusion, participants had between 5 and 35 reports (*M* = 24, *SD* = 7.43), for a total of 528 reports.

***ESM Measures***

In each survey, participants reported on their momentary feelings of autonomy (described below), along with other measures that were not relevant to this project.

**Single-Item** **Autonomy Measure.** We included the same single-item measure of autonomy used in Study 1, but replaced “In this situation” with “During the last hour,” for consistency with the timeframe used in the rest of the ESM survey. Specifically, participants were asked “During the last hour, were you free to behave however you wanted?” (1 = *Not a lot*, 5 = *Very much*).

**Autonomy Subscale from the Need Satisfaction Scale.** To validate our single-item autonomy measure, we included the three-item subscale of the Need Satisfaction Scale (La Guardia et al., 2000). This scale has been used in previous daily life research examining momentary autonomy (e.g., Weinstein et al., 2023) and has shown to have acceptable validity and reliability (La Guardia et al., 2000; Sağkal & Özdemi̇r, 2019). We reworded each item to refer to experiences during the past hour. Each item was preceded with “During the last hour,” and the three items were “I felt free to be who I am,” “I felt controlled or pressured to be certain ways,” and “I had a say in what happened, and I could voice my opinion” (ω_WP_ = .68; ω_BP_ = .86). Participants responded on a 7-point scale (1 = *Not at all true*, 7 = *Very true*).

***Data Analyses***

All analyses were conducted in RStudio (Version 4.3.1; R Core Development Team, 2020). The data had a nested structure, such that ESM reports (Level 1) were nested within participants (Level 2). First, we examined the within-person correlations among the four autonomy items, as well as between our one-item measure and the established three-item composite. Second, we ran a multi-level confirmatory factor analysis (MCFA) using the *lavaan* package in R (Rosseel, 2012) to test whether our new item and the established three items load onto the same latent construct. For this MCFA, all four items loaded onto a between-person factor and a within-person factor. However, because only the within-person factor loadings are relevant to our current research question, we only report the within-person loadings below.

**Results**

**Within-Person Correlations.** As shown in Table S1, our one-item autonomy measure correlated between .29 to .60 with each of the three items from the established autonomy subscale. The especially high correlation with the “I felt free to be who I am” item is likely due in part to content overlap. However, our one-item autonomy measure correlates an average of *r* = .37 with the other two established items, which is descriptively similar to the average inter-item correlation among the three established items (*r* = .34). We also observed a strong within-person correlation between the 3-item subscale composite and our 1-item autonomy measure, *r* = .60, 95% CI [.56, .65], *p* < .001.

**Table S1**

*Within-person correlations between 1-item measure and 3-item BPNS autonomy subscale and Standardized Within-Person Factor Loadings*

|  | Within-Person Correlations | | |  | Factor Loading |
| --- | --- | --- | --- | --- | --- |
|  | 1. | 2. | 3. |  |  |
| 1. Were you free to behave however you wanted? |  |  |  |  | .652 |
| 2. I felt free to be who I am. | .60 |  |  |  | 1.049 |
| 3. I felt controlled or pressured to be certain ways. [r] | .29 | .37 |  |  | .524 |
| 4. I had a say in what happened, and I could voice my opinion. | .45 | .47 | .15 |  | .659 |

*Note*. Item 1 is our new one-item measure of autonomy. Items 2–4 are from the autonomy subscale of the Basic Psychological Needs Scale. All items were preceded with the stem, “During the past hour.” Item 3 was reverse-scored prior to analyses.

**Multi-level Confirmatory Factor Analysis.** Next, we fit a single-factor MCFA to examine whether the four autonomy items loaded onto the same single latent autonomy construct. The single-factor model demonstrated good overall fit based on standard benchmarks suggested by Hu and Bentler (1999; CFI > .90; RMSEA < .06; SRMR < .08), CFI = .986, RMSEA = .055, SRMR = .030). As shown in Table S1, the within-person loading of our one-item measure was comparable to the loadings of two out of three items in the established three-item measure. Note that the standardized loading for the remaining item was higher than 1. We believe this is likely due to convergence issues with our relatively small sample size.

**Conclusion**

Providing strong evidence for the convergent validity of our one-item measure, we show that our one-item measure correlates strongly with an established three-item autonomy composite and loads strongly onto the same latent factor as these three established items.

# 2. Extended Interaction Partner Comparisons

**Table S2**

*Multilevel Models Predicting Autonomy from Interaction Partner*

|  |  |  |  | 95% CI | |
| --- | --- | --- | --- | --- | --- |
| Variable | *β* | SE | *p* | Lower | Upper |
| Comparisons with being alone |  |  |  |  |  |
| Romantic vs. Alone | 0.23 | 0.10 | .010 | 0.06 | 0.39 |
| Non-romantic vs. Alone | –0.29 | 0.04 | < .001 | –0.37 | –0.22 |
| Friends/Family vs. Alone | 0.01 | 0.05 | .741 | –0.07 | +0.09 |
| Others vs. Alone | –0.69 | 0.05 | < .001 | –0.77 | –0.61 |
| Comparisons between interaction partners |  |  |  |  |  |
| Romantic vs. Friends/Family | 0.13 | 0.08 | .074 | –0.01 | +0.27 |
| Romantic vs. Non-close others | 0.92 | 0.10 | < .001 | +0.75 | +1.09 |
| Friends/Family vs. Non-close others | 0.73 | 0.04 | < .001 | +0.66 | +0.80 |
| Friends vs. Family | –0.01 | 0.08 | .847 | –0.14 | +0.12 |

*Note*. For each analysis, the category listed first under “Variable” was coded 1, while the latter category was coded 0. For example, for the Romantic vs. Friends/Family variable, 1 = interaction involved only romantic partners, 0 = interaction involved only friends and family, and NA = all other moments (i.e., alone, multiple interaction partner types, or no interaction partner type selected). “Non-close others” = moments in which participants indicated that they were socially interacting, but did not select any of the partner types in the survey (i.e., friends, family, or romantic partner). “Non-Romantic” = moments included in “Non-close others” as well as interactions with friends and family. CI = Confidence Interval. Only partnered individuals were included in analyses that involved comparisons with romantic partners.

**Figure S1**

*Distributions of Autonomy by Interaction Partner Type*

*Note.* These violin plots illustrate the distribution of person-level means for each interaction partner type. For boxplots, the black horizontal line depicts the median and the white boxes depict the 25^th^ to 75^th^ Interquartile Range. Means and 95% confidence intervals are depicted in red. See Table S2 for significance tests of comparisons between partner types.

**References**

Hu, L., & Bentler, P. M. (1999). Cutoff criteria for fit indexes in covariance structure analysis: Conventional criteria versus new alternatives. *Structural Equation Modeling: A Multidisciplinary Journal*, *6*(1), 1–55. https://doi.org/10.1080/10705519909540118

La Guardia, J. G., Ryan, R. M., Couchman, C. E., & Deci, E. L. (2000). Within-person variation

in security of attachment: a self-determination theory perspective on attachment, need

fulfillment, and well-being. *Journal of Personality and Social Psychology*, *79*(3), 367. https://doi.org/10.1037/0022-3514.79.3.367

R Core Team (2020). R: A language and environment for statistical computing. R Foundation

for Statistical Computing, Vienna, Austria. https://www.R-project.org/.

Rosseel Y (2012). “lavaan: An R Package for Structural Equation Modeling.” *Journal of*

*Statistical Software*, *48*(2), 1–36. https://doi.org/10.18637/jss.v048.i02

Sağkal, A. S., & Özdemi̇R, Y. (2019). Need Satisfaction, Partner Attachment, and Romantic Relationship Quality: A Multiple Mediation Model. *Anadolu Journal of Educational Sciences International*, *9*(1), 163–180. https://doi.org/10.18039/ajesi.520839

Weinstein, N., Vuorre, M., Adams, M., & Nguyen, T. (2023). Balance between solitude and socializing: Everyday solitude time both benefits and harms well-being. *Scientific Reports*, *13*(1), Article 1. https://doi.org/10.1038/s41598-023-44507-7
